# Prognostic Value of the Lung Immune Prognostic Index on Recurrence after Radical Surgery for High-Risk Renal Cell Carcinoma

**DOI:** 10.3390/cancers16040776

**Published:** 2024-02-14

**Authors:** Yudai Ishiyama, Tsunenori Kondo, Kazuhiko Yoshida, Junpei Iizuka, Toshio Takagi

**Affiliations:** 1 Department of Urology, Tokyo Women’s Medical University, 8-1 Kawada-cho, Shinjuku-ku, Tokyo 162-0054, Japan; 2 Department of Urology and Transplant Surgery, Toda Chuo General Hospital, 1-19-3 Honmachi, Toda-shi, Saitama 335-0023, Japan; 3 Department of Urology, Tokyo Women’s Medical University Adachi Medical Center, 4-33-1 Kouhoku, Adachi-ku, Tokyo 123-8558, Japan

**Keywords:** inflammation, kidney cancer, nephrectomy, recurrence, tumor metabolism

## Abstract

**Simple Summary:**

Further risk stratification among high-risk renal cell carcinomas is relevant as it helps balance the benefits and drawbacks of adjuvant immunotherapy. The effects of the lung immune prognostic index (LIPI), which is calculated based on the derived neutrophil-to-lymphocyte ratio and lactate dehydrogenase levels, and was evaluated using a retrospective, multi-institutional database. Of the 235 patients with high-risk renal cell carcinoma (≥pT3 or N1–2 and M0), 119 (50.6%), 91 (38.7%), and 25 (10.6%) were categorized as good (0), intermediate (1), and poor (2) based on the LIPI score, respectively, and recurrence-free survival (RFS) was significantly correlated with the score groups (median progression-free survival: 90.8 vs. 21.2 vs. 10.0 months). The LIPI was an independent predictor of RFS, and prediction accuracy improved with the addition of the LIPI to preexisting scores. The LIPI can be a useful biomarker for predicting recurrence, particularly for identifying the highest-risk cohorts.

**Abstract:**

With emerging options in immediate postoperative settings for high-risk renal cell carcinoma (hrRCC), further risk stratification may be relevant for informed decision making. Balancing the benefits and drawbacks of adjuvant immunotherapy is recommended. We aimed to evaluate the effects of the lung immune prognostic index (LIPI) in this setting. This bi-institutional retrospective study recruited 235 patients who underwent radical surgery for hrRCC between 2004 and 2021. LIPI scores were calculated based on the derived neutrophil-to-lymphocyte ratio and lactate dehydrogenase levels. The association between LIPI scores and local or distant recurrence was analyzed, along with other possible clinical factors. The median recurrence-free survival (RFS) period was 36.4 months. Based on the LIPI scores, 119, 91, and 25 patients were allocated to the good, intermediate, and poor groups, respectively. The RFS was significantly correlated with the LIPI scores, and the 36 month survival rates were 67.3, 36.2, and 11.0% in the good, intermediate, and poor groups, respectively. In the multivariate model, the LIPI independently predicted the RFS, along with symptoms at diagnosis, Eastern Cooperative Oncology Group performance status, pT status, pN status, and tumor grade. The C-index of the LIPI in predicting RFS was 0.63, and prediction accuracy improved with the addition of the LIPI to both GRade, Age, Nodes, Tumor, and the UCLA Integrated Staging System. Conclusively, the LIPI can be a significant prognostic biomarker for predicting hrRCC recurrence, particularly for identifying the highest-risk cohort.

## 1. Introduction

The risk of recurrence of localized renal cell carcinoma (RCC) varies according to stage, with relatively low reported rates for stage 1 disease [1]. Most previous prediction models for RCCs targeted this disease as a whole and mainly comprised early-stage malignancies [2,3]. The idea of administering systemic therapy in adjuvant settings solely for high-risk RCCs (hrRCCs) has recently emerged, led by multiple randomized controlled phase III trials (RCTs) with a focus on immune checkpoint inhibitors (ICIs) [4]. The definition of hrRCC is not concrete, but it commonly encompasses pathological (p) T3–4 and nodal positivity (pN+). The prognosis for this patient group (pT3–4 or pN+) is distinct from earlier stage disease in which the 5 year recurrence rates exceed 40% [4]. KEYNOTE-564 is a landmark trial for patients with hrRCC in which pembrolizumab prolonged the disease-free survival, compared to that with placebo, for the first time in an adjuvant setting for any drug type [5]. However, whether to offer ICIs to patients who meet the RCT eligibility criteria remains debatable. Reports have indicated that more than 20% of patients in the KEYNOTE-564 trial discontinued treatment because of adverse events, including irreversible events, and that the treatment effect of pembrolizumab might not be the same for all risk categories [5]. Under such conditions, further risk stratification for hrRCC may facilitate shared decision making between patients and clinicians.

There is still room for the assessment of clinical biomarkers for the prediction of hrRCC. We have previously reported that C-reactive protein (CRP), which has already been utilized in various RCC settings, is a potential biomarker [6]. More recently, our interest has shifted to include the lung immune prognostic index (LIPI) score as a possible candidate. This score combines the derived neutrophil-to-lymphocyte ratio (dNLR), calculated based on white cell counts and neutrophils only, with the levels of lactate dehydrogenase (LDH), a commonly measured laboratory marker for RCC management [7,8]. Evidence on the utility of LIPI is accumulating, mainly in immunotherapy for malignancies outside of RCC [9,10,11], and emerging data suggest its potential role in RCC or postsurgical prognosis prediction [10,12,13,14].

Therefore, in this study, we aimed to verify the prognostic value of the LIPI for hrRCC recurrence.

## 2. Materials and Methods

### 2.1. Patient Selection and Data Collection

The study protocol was approved by the Internal Ethics Review Board of Tokyo Women’s Medical University (approval ID: 2020-0062) and was conducted in accordance with the Declaration of Helsinki. The requirement for obtaining formal consent was waived owing to the retrospective and observational nature of this study. All data were extracted from medical records.

We conducted a retrospective study of patients diagnosed with nonmetastatic, high-risk renal cell carcinoma (RCC) who underwent curative surgery at two tertiary care centers affiliated with Tokyo Women’s Medical University from January 2004 to August 2021. The high-risk criteria were based largely on the inclusion criteria from the KEYNOTE-564 study, specifically pT3 or higher staging, or pN1-2 lymph node involvement confirmed histopathologically [15]. For patients who had enlarged regional lymph nodes in the pre-surgical imaging, only those with their nodes subsequently completely resected were included. Out of 307 initially recruited patients, those with inadequate laboratory data (n = 54) or incomplete follow-up (n = 18) were excluded. Consequently, 235 patients were eligible for the final analysis. Fuhrman grade [16] was used for tumor grading instead of the World Health Organization/International Society of Urologic Pathology grade [17], owing to the period of patient enrollment.

### 2.2. Surgery and Follow-Up Protocol

All patients in this study underwent a radical nephrectomy, performed either as an open or laparoscopic procedure, in accordance with the guidelines set forth in Hinman’s Atlas of Urologic Surgery [18,19,20]. Preoperative planning included an enhanced computed tomography (CT) scan administered at least three weeks prior to surgery to ensure accurate clinical staging. Postoperatively, regular follow-up assessments were conducted, including imaging and laboratory tests at intervals of every three to six months. Additional scans were performed as needed, based on the individual clinical requirements of each patient. No patients in this study were treated with adjuvant systemic therapy.

### 2.3. Study Design and Statistical Analyses

The main outcome of interest in this study was recurrence-free survival (RFS), defined as the time from surgery to either (1) first local or distant recurrence or metastasis or (2) death, whichever occurred first. Patients without documented recurrence or death at the final follow-up were censored. RFS was estimated using the Kaplan–Meier method and compared using the log-rank test.

**Figure 1:**
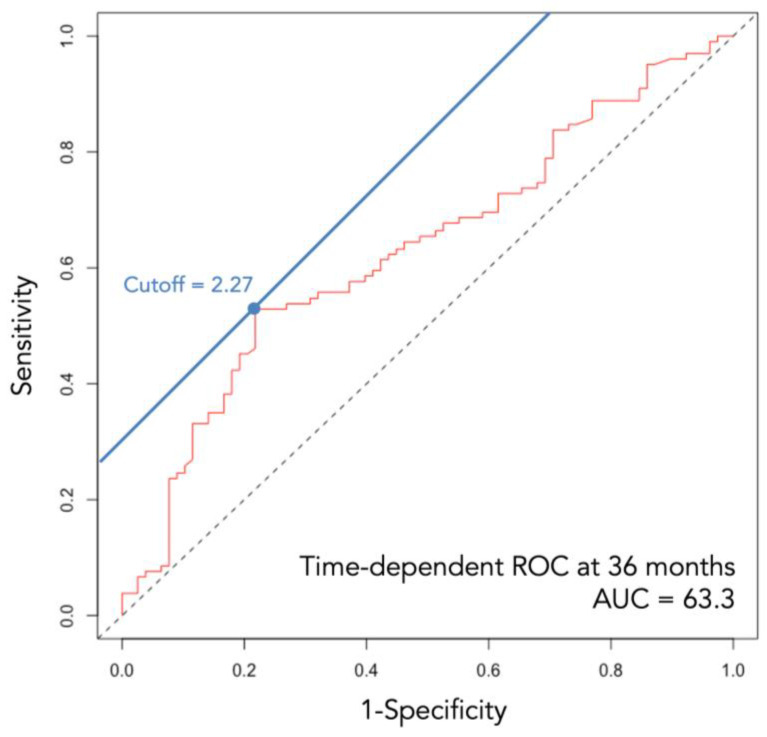
Time-dependent ROC curve at 36 months. AUC, area under curve; ROC, receiver operating characteristic.

The LIPI was calculated based on the dNLR and LDH values evaluated within one month before surgery. The dNLR was calculated using the following formula: (number of white blood cells)/(number of neutrophils). The cutoff value was set at 2.27, based on the receiver operating characteristic (ROC) curve analysis (Figure 1).

For LDH, we used the upper normal limit (UNL; 222 IU/L) as a reference point, according to previous literature [9,10,11]. The study cohort was divided into three composite score groups based on the LIPI scores as follows: good (0), intermediate (1), and poor (2), and RFS was compared among the groups. Moreover, the LIPI; its components (dNLR and LDH); and other candidates for predictors of RFS were analyzed using univariate and multivariate Cox proportional hazard regression models. The variables considered included age, sex, symptom presence at diagnosis, Eastern Cooperative Oncology Group performance status (ECOG-PS), tumor size, pT stage, pN stage, tumor pathology (clear cell or non-clear cell), and tumor grade. The associated risks were quantified as hazard ratios (HRs) with 95% confidence intervals (CIs). The dNLR and LDH were excluded from the multivariate model as they were components of the LIPI score. We did not include CRP as a possible variable because of data unavailability from the complete case analysis design. The accuracy of LIPI in RFS prediction was assessed using Harrell’s concordance index (C-index), and internal validation with 200 bootstrap samples was conducted to assess optimism. Furthermore, the C-index was compared before and after the addition of the LIPI to preexisting prediction scores (UCLA Integrated Staging System [UISS] model and GRade, Age, Nodes, and Tumor [GRANT] score) [21,22]. Continuous variables were analyzed using the Mann–Whitney U-test, and categorical variables were analyzed using the chi-squared or Fisher’s exact test with 95% CIs. All analyses were performed using the R software (The R Foundation for Statistical Computing, Vienna, Austria). Statistical significance was set at *p* < 0.05. This study was conducted using a complete case analysis.

## 3. Results

### 3.1. Patient Characteristics

Patient characteristics are summarized in Table 1. The median age of the overall cohort was 67.0 (61.0–73.0] years, and analysis showed no significant variance in the age distribution among the three prognosis groups categorized by the LIPI score: good (119 participants, 50.6%), intermediate (91 participants, 38.7%), and poor (25 participants, 10.6%). There were significant differences in the distribution of symptoms at diagnosis, with a trend toward more patients being positive in the order of good, intermediate, and poor (*p* = 0.011), whereas no such differences were observed for sex or ECOG-PS. The tumor size was significantly larger in the poor group than in the intermediate or good groups (*p* = 0.014). Regarding the histopathological profile, the distribution of the pT stage was significantly different among the three groups, with a higher prevalence of pT1/2, in the order of good, intermediate, and poor (*p* < 0.001). There was a significant difference in the proportion of patients with non-clear cell pathology, with the largest representation seen sequentially in the poor, intermediate, and good prognostic groups (*p* = 0.034), and there was a trend toward higher tumor grade in this order (*p* = 0.041). More patients with nodal-positive disease were included in the intermediate group (14.3%) than in the good group (8.4%), but not in the poor group (*p* = 0.080).

### 3.2. RFS According to LIPI Groups

**Figure 2:**
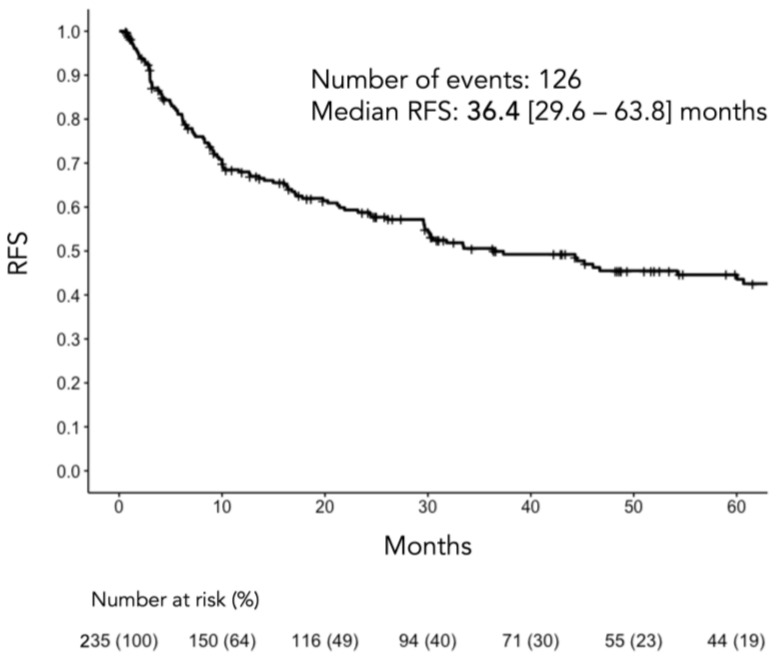
RFS of the entire study cohort. CI, confidence interval; RFS, recurrence-free survival.

During the median 19.8 (5.9–48.3) months of follow-up, 126 (53.6%) cases of recurrence occurred, with 47 (39.5%), 61 (67.0%), and 18 (72.0%) cases in the good, intermediate, and poor groups, respectively (*p* < 0.001). The median RFS period for the entire cohort was 36.4 (29.6–63.8) months (Figure 2).

**Figure 3:**
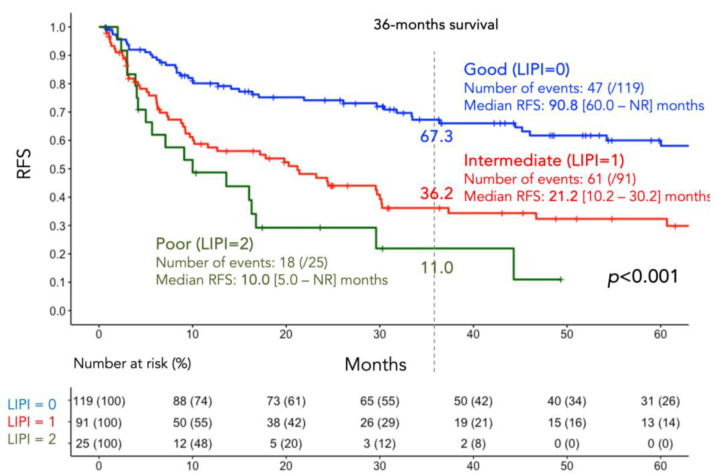
RFS according to the three LIPI score groups. CI, confidence interval; NR, not reached; RFS, recurrence-free survival.

RFS was significantly shorter in the poor group (median, 10.0 [95% CI, 5.0–not reached [23] months) compared to that in the intermediate 21.2 [95% CI, 0.2–30.2] and good 90.8 [95% CI, 60.0NR] groups (*p* < 0.001) (Figure 3).

At the landmark of 36 months, the survival rates were 67.3%, 36.2%, and 11.0% for the good, intermediate, and poor groups, respectively, and at 60 months, all patients in the poor group were either censored or died (61.4 34.9).

### 3.3. Factors Associated with RFS

Univariate analysis revealed that the presence of symptoms at diagnosis (HR: local, 2.27; systemic, 4.34 in reference to none; *p* < 0.001), ECOG-PS (HR, 1.67 for ≥1 in reference to 0; *p* = 0.025), tumor size (HR, 1.01 per mm; *p* = 0.001), pT (HR for T3a, 2.43; T3b, 3.64; T3c, 5.78, T4, 5.97 in reference to T1/T2; all *p* = 0.001 or lower), pN (HR, 2.28 for N1 in reference to N0/Nx; *p* = 0.002), tumor grade (HR for 3, 3.28; 4, 6.48 in reference to 1; both *p* < 0.05), histology (HR, 1.84 for non-clear cell in reference to clear cell; *p* = 0.008), LIPI score (HR for intermediate, 2.32; poor, 3.76 in reference to good; both *p* < 0.001), dNLR (HR, 1.23 per unit; *p* = 0.001; HR, 2.14 for ≥2.28 in reference to <2.28; *p* < 0.001), and LDH level (HR, 1.74 per unit; *p* < 0.001; HR, 2.13 for ≥UNL in reference to <UNL; *p* < 0.001) were significantly associated with RFS, whereas age and sex were not. Multivariate analyses further revealed that the presence of symptoms, ECOG-PS < pT, pN, tumor grade (grade 4 only), and LIPI score independently predicted RFS (Table 2).

### 3.4. Additive Role of the LIPI to Preexisting Prediction Models

**Figure 4:**
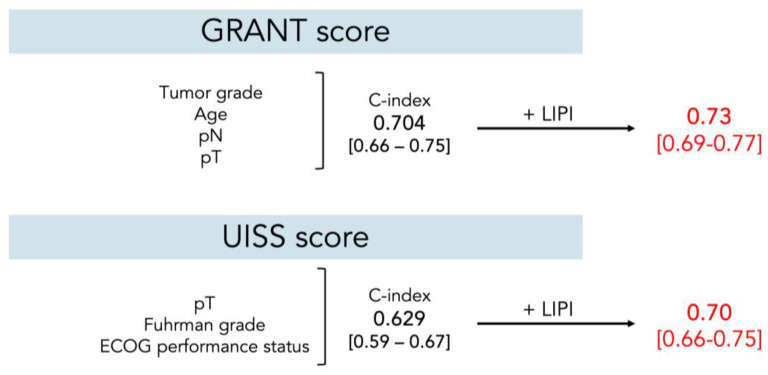
C-index before and after adding the LIPI to preexisting scores. C-index: Harrell’s concordance index; ECOG-PS, Eastern Cooperative Oncology Group performance status; GRANT, GRade, Age, Nodes, and Tumor; UISS, UCLA Integrated Staging System.

The C-index of the LIPI as a single score was 0.63 (0.58–0.68). Internal validation using bootstrapping revealed that optimism-corrected C-index was 0.63 (0.62–0.63). The C-index for the GRANT score (tumor grade, age, pN, pT) in this cohort was 0.70 (0.66–0.75). With the addition of LIPI as the fifth covariable, the value increased to 0.73 (0.69–0.77). Similarly, the UISS model (pT, tumor grade, ECOG-PS) scoring showed the accuracy C-index of 0.63 (0.59–0.67), and with the addition of the LIPI, the value increased to 0.70 (0.66–0.75) (Figure 4).

## 4. Discussion

In the present study, which included patients with hrRCC, disease recurrence occurred in 53.6% of the patients during a median follow-up of 20 months. A significantly shorter RFS was observed in the high LIPI score group. The LIPI was able to independently predict the RFS with an HR of 2.73 in the poor group. When appended to two major preexisting prediction models, the LIPI showed a clinically significant improvement in the C-index. In summary, the LIPI showed its potential as a prognostic biomarker for recurrence after radical resection of hrRCC.

Currently, two major factors render RFS prediction in hrRCC clinically relevant and important. First, although pembrolizumab has proven beneficial in reducing recurrence risk, its advantages must be discussed along with the potential harm of the occurrence of immune-related adverse events. Current guidelines recommend that an extremely careful, shared decision making process must be applied when considering adjuvant therapy in the absence of overall survival data and with the inconsistent results of other immunotherapy trials [24,25,26,27,28]. They also state that a search for biomarkers is warranted to identify patients who respond to therapy [28]. Second, detecting recurrence at an early stage is important in RCC, particularly because the benefits of metastasectomy have been reported. Available data suggest that the number of metastatic sites and the complete resection of all metastatic lesions are important prognosticators [29,30,31].

In this study, we specifically emphasized the LIPI. In one of the earliest studies, pretreatment LIPI was correlated with outcomes in ICIs for non-small cell lung cancer, but not in chemotherapy, suggesting that this score may be specific for certain settings [9]. Its prognostic value has already been demonstrated in other tumor types in ICI settings, including RCC [10]. In fact, recent post hoc analyses revealed that LIPI correlates with outcomes of metastatic RCC treated with immune checkpoint inhibitors and tyrosine kinase inhibitors [13,14]. The LIPI encompasses two types of biomarkers, dNLR and LDH, each possessing a strong rationale. dNLR (and more frequently NLR) has been extensively studied to reflect the inflammatory response to cancer [32]. Moreover, LDH reflects tumor metabolism, including enhanced glycolytic activity and necrosis due to hypoxia, and is reported to heavily correlate with ICI treatment response or RCC outcomes [33,34]. Therefore, it is reasonable to assume that the LIPI also plays a role in RCC, a tumor frequently linked to inflammatory activity [35].

In the present study, we showed, for the first time, that the LIPI could predict outcomes of patients with RCC in a postsurgical setting. The utility of the LIPI beyond the ICI setting has been poorly clarified; one rare example is a report on radical cystectomy, and this study adds to the current body of literature [12]. In the current cohort, approximately 10% of the patients were categorized into the poor LIPI group, and their prognosis was extremely poor. Furthermore, the outcomes of patients in the intermediate group (approximately 38%) were also far from satisfactory compared to the acceptable survival rate shown in the good group. Therefore, we argue that individuals who meet either of the criteria, dNLR or LDH, are possible candidates for adjuvant therapy or even stricter follow-up. Integrating the LIPI with other scores or prediction models is another viable option for the clinical application demonstrated in this study, given the significant strength of the LIPI in its clinical availability. Both dNLR and LDH are routinely measured in daily clinics, especially in RCC cases, considering their established roles in advanced cases [36,37]. The crucial next step involves identifying the LIPI model with the optimal performance, which we reluctantly desisted largely due to a lack of information on necrosis in pathological reports. These reports were only recently standardized in Japanese pathologic reporting. Moreover, future directions will include investigating the LIPI for its potential in differentiating patients benefiting from ICIs used as adjuvant therapy, based on the previously mentioned phenomenon that this score may be specific for certain settings, potentially involving ICIs [9].

Issues regarding the cutoff values for the dNLR and LDH are noteworthy, as reports are inconsistent. For dNLR, cutoff values of 3.0 and 3.8 have been reported, along with 2.29 in our previous study on pembrolizumab for urothelial carcinoma [32,38,39]. We adopted the original value, 2.28, for this cohort according to the ROC curve analysis, to be consistent with the aforementioned study [39]. For LDH, the reports have been rather conclusive to support the use of the upper limit of each laboratory’s reference. However, the optimal value remains debatable.

The current study is subject to several limitations. The retrospective design and the exclusive enrollment from tertiary care centers may introduce selection bias. Due to the unavailability of data, we did not consider specific variant histologies or aberrant growth patterns, such as sarcomatoid features or necrosis, despite their reported prognostic significance [40,41,42]. Cancer-specific and overall survival outcomes were not evaluated, based on the presumption that recent advancements in systemic treatments for recurrent disease would significantly alter these outcomes, thereby rendering our assessments potentially outdated [43]. Furthermore, no comparative analysis was conducted with other inflammatory markers such as NLR and CRP, despite their established prognostic value, due to the absence of comprehensive data [44]. Lastly, the findings of this study have not been externally validated with an independent cohort, which is necessary to corroborate our results definitively.

## 5. Conclusions

LIPI can be a significant prognostic biomarker for recurrence prediction in hrRCC, particularly when identifying the highest-risk cohort. Utilizing the LIPI may aid in decision making regarding adjuvant therapy and vigilant observation.

## Figures and Tables

**Table 1:** Patient characteristics.

	Overall 235	Good LIPI = 0 119 (50.6%)	Intermediate LIPI = 1 91 (38.7%)	Poor LIPI = 2 25 (10.6%)	*p*
Age, years (median [IQR])	67.0 [61.0–73.0]	67.0 [61.5–74.0]	66.0 [61.5–72.0]	69.0 [57.0–74.0]	0.915
Sex, male (%)	166 (70.6)	84 (70.6)	65 (71.4)	17 (68.0)	0.946
ECOG-PS (%)					0.359
0	196 (83.4)	102 (85.7)	72 (79.1)	22 (88.0)	
≥1	39 (16.6)	17 (14.3)	19 (20.9)	3 (12.0)	
Symptoms at diagnosis	104 (44.3)	65 (54.6)	31 (34.1)	8 (32.0)	0.011
None	95 (40.4)	42 (35.3)	43 (47.3)	10 (40.0)	
Local	36 (15.3)	12 (10.1)	17 (18.7)	7 (28.0)	
Systemic	104 (44.3)	65 (54.6)	31 (34.1)	8 (32.0)	
Tumor size, mm (median [IQR])	77.0 [59.0–100.0]	74.5 [57.0–90.0]	85.0 [61.5–107.0]	91.0 [65.0–113.0]	0.014
Pathological T stage (%)					<0.001
T1/T2	62 (26.4)	42 (35.3)	17 (18.7)	3 (12.0)	
T3a	101 (43.0)	57 (47.9)	35 (38.5)	9 (36.0)	
T3b	47 (20.0)	11 (9.2)	26 (28.6)	10 (40.0)	
T3c	20 (8.5)	8 (6.7)	9 (9.9)	3 (12.0)	
T4	5 (2.1)	1 (0.8)	4 (4.4)	0 (0.0)	
pN stage (%)					
N0/Nx	212 (90.2)	109 (91.6)	78 (85.7)	25(100.0)	0.080
N1	23 (9.8)	10 (8.4)	13 (14.3)	0 (0.0)	
Pathological histology (%)					0.034
Clear cell	202 (86.0)	108 (90.8)	76 (83.5)	18 (72.0)	
Non-clear cell	33 (14.0)	11 (9.2)	15 (16.5)	7 (28.0)	
Tumor grade (%)					0.041
1	16 (6.8)	10 (8.4)	5 (5.5)	1 (4.0)	
2	97 (41.3)	57 (47.9)	32 (35.2)	8 (32.0)	
3	89 (37.9)	44 (37.0)	34 (37.4)	11 (44.0)	
4	33 (14.0)	8 (6.7)	20 (22.0)	5 (20.0)	
Recurrence	126 (53.6)	47 (39.5)	61 (67.0)	18 (72.0)	<0.001
Follow-up periods, months	19.8 [5.9–48.3]	31.8 [9.4–62.7]	12.6 [3.8–30.5]	9.1 [4.0–16.8]	<0.001

Data are expressed as n (%) or medians (interquartile ranges). BMI, body mass index; ECOG-PS, Eastern Cooperative Oncology Group performance status; IQR, interquartile range; LIPI, lung immune prognostic index.

**Table 2:** Univariate and multivariate analyses of possible factors associated with RFS.

	Univariate			Multivariate		
	HR	95% CI	*p*	HR	95% CI	*p*
Age (continuous)	1.00	0.99–1.02	0.837	1.00	0.98–1.02	0.836
Sex						
Male				Ref		Ref
Female	1.01	0.69–1.49	0.953	0.86	0.56–1.33	0.503
Presence of symptoms						
None				Ref		
Local	2.27	1.50–3.43	0.000	2.06	1.31–3.23	0.002
Systemic	4.34	2.65–7.11	0.000	2.19	1.26–3.82	0.006
ECOG-PS						
0				Ref		
≥1	1.67	1.07–2.60	0.025	1.65	1.00–2.71	0.049
Tumor size (continuous)	1.01	1.00–1.01	0.001	1.01	1.00–1.01	0.062
pT						
T1/T2	Ref			Ref		
T3a	2.43	1.45–4.07	0.001	2.26	1.30–3.91	0.004
T3b	3.64	2.08–6.36	0.000	2.37	1.25–4.51	0.009
T3c	5.78	2.90–11.56	0.000	4.57	2.14–9.75	<0.001
T4	5.97	2.03–17.56	0.001	5.80	1.76–19.10	0.004
pN						
N0/Nx				Ref		
N1	2.28	1.37–3.82	0.002	4.05	2.09–7.84	<0.001
Tumor grade						
1	Ref			Ref		
2	1.66	0.66–4.19	0.286	1.35	0.51–3.54	0.549
3	3.28	1.31–8.26	0.011	2.43	0.92–6.44	0.074
4	6.48	2.45–17.16	0.000	2.94	1.05–8.25	0.040
Histology						
Clear cell	Ref			Ref		
Non-clear cell	1.84	1.18–2.89	0.008	1.30	0.75–2.26	0.349
LIPI						
Good (0)	Ref			Ref		
Intermediate (1)	2.32	1.58–3.41	0.000	1.59	1.04–2.44	0.033
Poor (2)	3.76	2.15–6.57	0.000	2.73	1.41–5.28	0.003
dNLR						
Continuous	1.23	1.09–1.38	0.001	NA		
≥2.28 (Ref: <2.28)	2.14	1.50–3.06	0.000	NA		
LDH						
Continuous	1.74	1.39–2.19	0.000	NA		
≥UNL (Ref: <UNL)	2.13	1.45–3.14	0.000	NA		

Data are expressed as hazard ratios (HRs) and 95% confidence intervals (CIs). BMI, body mass index; dNLR, derived neutrophil-to-lymphocyte ratio; ECOG-PS, Eastern Cooperative Oncology Group performance status; IQR, interquartile range; LDH, lactate dehydrogenase; LIPI, lung immune prognostic index; NA, not applicable; UNL; upper normal limit; Ref, reference; RFS, recurrence-free survival.

## Data Availability

The data are available from the corresponding author (Y.I.) upon reasonable request.
